# Supplementary material for: Feasibility of a serious game coupled with a contact-based session led by lived experience workers for depression prevention in high-school students
Source: PLoS One. 2021 Nov 30;16(11):e0260224. doi: 10.1371/journal.pone.0260224 (PMC8631635; doi:10.1371/journal.pone.0260224)
Supplement: Attachment — Submitted filename: Reply reviewer_PLOS_20211101.docx [file pone.0260224.s004.docx]

Mandy Gijzen
Trimbos Institute
P.O. Box 725, 3500 AS, Utrecht,

The Netherlands.

01-11-2021

Response to the Editor and the Reviewer

Ref: PONE-D-21-17344R1

Title: Feasibility of a serious game coupled with a contact-based session led by lived experience workers for depression prevention in high-school students

Journal: PLOS ONE

Dear Dr. Mordaunt,

We would like to thank you for the opportunity to respond to the comments of reviewer 3 to improve and further clarify our manuscript. The comments of the editor in italics; followed by our response, while changes made in the manuscript are between quotation marks.

*1. The study appears to present the results of original research. It appears to be an implementation/scaling of an approach developed in an earlier clinical trial.
2. Results do not appear to have been published elsewhere. The second reviewer has raised the question of "salami slicing" and it would be helpful for the authors to clarify what approach has been taken with regards to the publication of results from the underlying study. My interpretation is that this could be considered an "extension phase" of the original trial, or as the authors have called it, an implementation study focused on the scaling of the capability developed and studies in the original trial. I do think implementation science is a separate issue to clinical trials, but I think clearly distinguishing this is important for the record.*

In regard to comments 1 and 2:

In short,

1. The preventive STORM program (consisting of several interventions) is currently being evaluated in an RCT
2. During the trial one preventive intervention -Moving Stories- proofed hard to execute as several obstacles were met during the trial.
3. This raised questions about the feasibility of rolling out Moving Stories in the future, once the STORM program effectiveness has been ascertained.
4. Hence the need for a **feasibility** study to look into the barriers and facilitating factors for implementing Moving Stories

Therefore, our study is

- **Not** an implementation study in the sense of STaRI (which has to be conducted once the interventions has been proven to be effective)
- **Neither** a hybrid implementation-evaluation study (with some amount of salami slicing on our part)
- **But a feasibility study** looking into the barriers and facilitating factors for Moving Stories’ future role out should STORM has been proven to be effective.

In our Abstract and Introduction we have added a clarification of the specific aim and intent of the study.

In the title of our MS we used terms to describe to study’s methodology: originally *Process Evaluation* and after revision *Feasibility of Scaled-up Implementation*. Now we begin to see that these qualifiers were unhelpful and may have caused confusion about the purpose of our study. Hence, we have changed the title of the MS once again (please, see below) and tried to do a better job in clarifying the purpose of the MS in both the Abstract and Introduction (please, see below).

**Changes made in manuscript:**

Title:

“Feasibility of a serious game coupled with a contact-based session led by lived experience workers for depression prevention in high-school students”

Abstract, page 2:

Lines 30-32:

“The current study was carried out to assess inhibiting and promoting factors for scaling up Moving Stories once its effectiveness has been ascertained.”

Lines 43-44:

“Bringing Moving Stories to scale in the high-school setting appears feasible, but will remain logistically somewhat challenging.”

Introduction, page 6, lines 118-127:

“Therefore, the primary aim of the current study was to assess the feasibility of implementing Moving Stories as a universal preventive intervention within the STORM program as this intervention was newly added within the STORM approach. More specifically, during the STORM trial we aimed to record (1) the number and percentage of high-school students that actively played Moving Stories (coverage rate), (2) the average number of playing days out of the five (participation rate), (3) how successful LEWs were in reviewing all five mental health first aid (MHFA) strategies during the debriefing sessions and to what extend students could identify depressive symptoms (fidelity), (4) what other themes were addressed and resonated with the students during the debriefing sessions, and how the students responded to the personal experiences of the LEWs.”

Methods, page 12, line 255:

“Data was collected during the STORM trial.”

*3. Experiments, statistics, and other analyses are largely performed to a high technical standard and are described in sufficient detail. The second reviewer has raised the possibility of whether aspects such as the interviews being recorded with notes rather than with recordings/coding, constitute a critical flaw. In the context of this being an implementation study, I would put it to the authors to respond to this with regards to feasibility in particular. It has been commented on but given that we could anticipate that this might be a criticism post-publication, this may warrant expanding on.*

We have added a clarification on the chosen method of recording in our Methods.

**Changes made in manuscript:**

Methods, Data collection, page 12-13, lines 275-279:

“In total, 49 debriefing sessions were provided by 11 LEWs. Notes were taken of 18 debriefing sessions to record what the LEW and students discussed during the debriefing sessions: 8 during study year of 2017-2018 and 10 during 2018-2019 with at least one debriefing session per school (see Table 1). These notes were kept by four researchers to ensure that topics noted were not the results of reporter bias.”

Discussion, Strengths and Limitations, pages 29, lines 672-675:

“Tape-recording would have legal implications as participants would be identifiable. Moreover, tape-recording has the disadvantage participants are less likely to discuss personal issues freely [52, 53].”

*4. Conclusions are presented in an appropriate fashion and are supported by the data.
5. The article is presented in an intelligible fashion and is written in standard English.
6. The research meets all applicable standards for the ethics of experimentation and research integrity.*

*7. The article adheres to appropriate reporting guidelines and community standards for data availability, however I would suggest that upon clarifying that this is an implementation study rather than an extension phase of a clinical trial, that the authors consider reviewing the manuscript for adherence to the Standards for Reporting Implementation Studies (StaRI) Statement (*[*https://www.equator-network.org/reporting-guidelines/stari-statement/*](https://www.equator-network.org/reporting-guidelines/stari-statement/)*). This is particularly helpful if the manuscript is later included in a systematic review or other form of meta-research, in to maximise the assessed quality of the study (not withstanding any critical problems).*

Please find the STaRI checklist, as requested

Once again, many thanks for allowing us to make these revisions.

On behalf of all authors,

Mandy Gijzen
